# Pixel-wise annotation for clear and contaminated regions segmentation in wireless capsule endoscopy images: A multicentre database

**DOI:** 10.1016/j.dib.2024.110927

**Published:** 2024-09-10

**Authors:** Vahid Sadeghi, Yasaman Sanahmadi, Maryam Behdad, Alireza Vard, Mohsen Sharifi, Ahmad Raeisi, Mehdi Nikkhah, Alireza Mehridehnavi

**Affiliations:** a Student Research Committee, School of Advanced Technologies in Medicine, Isfahan University of Medical Sciences, Isfahan, Iran; b Medical Image & Signal Processing Research Center, School of Advanced Technologies in Medicine, Isfahan University of Medical Sciences, Isfahan, Iran; c Department of Bioelectrics and Biomedical Engineering, School of Advanced Technologies in Medicine, Isfahan University of Medical Sciences, Isfahan, Iran; d Department of Electrical Engineering, Yazd University, Yazd, Iran; e Gastroenterologist & Hepatologist Fellowship of Endosonography Isfahan University of Medical Sciences, Isfahan, Iran; f Department of Internal Medicine, Clinical Research Development Unit, Hajar Hospital, Shahrekord University of Medical Sciences, Shahrekord, Iran; g Gastrointestinal and Liver Diseases Research Centre, Iran University of Medical Sciences, Tehran, Iran

**Keywords:** Bubble, Ground truth masks, Small bowel capsule endoscopy, Small bowel visualization quality, Turbid fluids

## Abstract

Wireless capsule endoscopy (WCE) is capable of non-invasively visualizing the small intestine, the most complicated segment of the gastrointestinal tract, to detect different types of abnormalities. However, its main drawback is reviewing the vast number of captured images (more than 50,000 frames). The recorded images are only sometimes clear, and different contaminating agents, such as turbid materials and air bubbles, degrade the visualization quality of the WCE images. This condition could cause serious problems such as reducing mucosal view visualization, prolonging recorded video reviewing time, and increasing the risks of missing pathology. On the other hand, accurately quantifying the amount of turbid fluids and bubbles can indicate potential motility malfunction. To assist in developing computer vision-based techniques, we have constructed the first multicentre publicly available clear and contaminated annotated dataset by precisely segmenting 17,593 capsule endoscopy images from three different databases.

In contrast to the existing datasets, our dataset has been annotated at the pixel level, discriminating the clear and contaminated regions and subsequently differentiating bubbles and turbid fluids from normal tissue. To create the dataset, we first selected all of the images (2906 frames) in the reduced mucosal view class covering different levels of contamination and randomly selected 12,237 images from the normal class of the copyright-free CC BY 4.0 licensed small bowel capsule endoscopy (SBCE) images from the Kvasir capsule endoscopy database. To mitigate the possible available bias in the mentioned dataset and to increase the sample size, the number of 2077 and 373 images have been stochastically chosen from the SEE-AI project and CECleanliness datasets respectively for the subsequent annotation. Randomly selected images have been annotated with the aid of ImageJ and ITK-SNAP software under the supervision of an expert SBCE reader with extensive experience in gastroenterology and endoscopy. For each image, two binary and tri-colour ground truth (GT) masks have been created in which each pixel has been indexed into two classes (clear and contaminated) and three classes (bubble, turbid fluids, and normal), respectively.

To the best of the author's knowledge, there is no implemented clear and contaminated region segmentation on the capsule endoscopy reading software. Curated multicentre dataset can be utilized to implement applicable segmentation algorithms for identification of clear and contaminated regions and discrimination bubbles, as well as turbid fluids from normal tissue in the small intestine.

Since the annotated images belong to three different sources, they provide a diverse representation of the clear and contaminated patterns in the WCE images. This diversity is valuable for training the models that are more robust to variations in data characteristics and can generalize well across different subjects and settings. The inclusion of images from three different centres allows for robust cross-validation opportunities, where computer vision-based models can be trained on one centre's annotated images and evaluated on others.

Specifications Table

**Table:** 

Subject	Biomedical Engineering
Specific subject area	Medical Image Processing, Computer Vision, Wireless Capsule Endoscopy, Gastroenterology, Clear and Contaminated Region Segmentation, Small Bowel, Artificial Intelligence (AI), Bubbles and Turbid Fluids.
Data format	1. RGB and PNG (.png) raw images with Resolution of 336×336 and 576×576 pixels 2. Black-and-white and JPG (.jpg) manually annotated binary masks 3. RGB and JPG (.jpg) manually annotated tri-colour masks
Type of data	Image
Data collection	The total number of 15,143, 2077, and 373 SBCE images with different contamination levels have been randomly chosen from the publicly existing Kvasir capsule endoscopy database (https://osf.io/dv2ag/), SEE-AI project database (https://www.kaggle.com/datasets/capsuleyolo/kyucapsule) and the CECleanliness dataset (https://cvblab.synology.me/PublicDatabases/CECleanliness/CECleanlinessValidation.zip), respectively . The mentioned datasets are licensed under Creative Commons Attribution 4.0 International (CC BY 4.0), which means copying, redistributing, reusing, sharing, and reproducing the database materials accessible in any medium or format. GT-annotated masks were produced using two open-source software (ImageJ [1] and ITK-SNAP [2]). Images in our curated dataset contain turbid fluids and bubbles in complex colours and textures with different amounts and spatial contamination distributions.
Data source location	Primary raw wireless capsule endoscopy image sources: 1. Kvasir-Capsule, a video capsule endoscopy dataset [3] 2. Small bowel capsule endoscopy examination and open access database with artificial intelligence: The SEE-artificial intelligence project [4] 3. Automatic evaluation of degree of cleanliness in capsule endoscopy based on a novel CNN architecture [5] Secondary annotated image source: 1. Department of Bioelectrics and Biomedical Engineering, School of Advanced Technologies in Medicine, Isfahan University of Medical Sciences, Isfahan, Iran, Latitude: 32.606929, Longitude: 51.651331.
Data accessibility	Repository name: Mendeley Data Data identification number: 10.17632/vmxhn95j8z.3 Direct URL to data: https://data.mendeley.com/datasets/vmxhn95j8z/3
Related research article	Vahid Sadeghi, Alireza Mehridehnavi, Yasaman Sanahmadi, Sajed Rakhshani, Mina Omrani, Mohsen Sharifi “Real-time small bowel visualization quality assessment in wireless capsule endoscopy images using different lightweight embeddable models” [6]

## 1 Value of the Data

• This is the first and only publicly available annotated dataset for evaluating the small bowel visualization quality (SBVQ) score.
• The curated database employed in this study consists of three different raw datasets designed for generalization and the development of models that exhibit robustness in the face of data diversity with different characteristics.
• Regarding the physiological meaning of bubbles and turbid fluids, the provided dataset can be used to evaluate intestinal motility disorders.
• The presented dataset can be used to compare the inter-observer agreement on SBVQ score prediction among three experienced endoscopist readers and the computer vision-based algorithms.
• The sizeable curated pixel-level annotated WCE image dataset serves as a powerful resource to develop AI-based techniques, particularly those reliant on substantial labelled data, for objective and quantitative evaluation of small bowel cleanliness in the WCE images.
• Provided quantitative Excel files can be utilized to turn the clear and contaminated region segmentation into a regression or classification problem.

## 2 Background

The lack of pixel-wise annotated datasets limits the development of AI-based techniques for clear and contaminated region segmentation. Most of the computer vision studies on clear and contaminated region localization, which have been published in recent years, either used non-publicly available datasets [[7], [8], [9]] or raw image databases [5]. There are several clinical applications that require publicly accessible datasets, including clear and contaminated region segmentation, localization of different types of small bowel contents (such as turbid fluids and bubbles) with different physiological meanings from normal mucosa, developing computer vision-based models to shorten the long time WCE videos by rejecting contaminated frames, and objective assessment of the SBVQ. The lack of a publicly accessible dataset for the mentioned functionalities motivated us to release a multicentre, comparatively large, publicly available, expert-supervised representative dataset of SBCE images.

## 3 Data Description

Our provided dataset is publicly available in a data repository [10]. In the constructed dataset, we provide material for segmenting contaminated regions from clear ones in WCE images and further discrimination between contaminated agents (turbid liquids and bubbles). In the Excel files, the percentage of the clear region in each individual frame, along with its cleanliness class, has been attached.

The inter-rater agreement among three gastroenterologists has been evaluated by analyzing a total number of 153 images randomly selected from the Kvasir, SEE-AI, and CECleanliness datasets. Each individual image underwent annotation by the three annotators. Each annotator carried out the annotation process under the supervision of one specialist, resulting in the creation of three masks per image. This approach facilitated a comprehensive assessment of agreement levels and provided insights into areas of consensus and discrepancy among the raters.

**Fig. 1:**
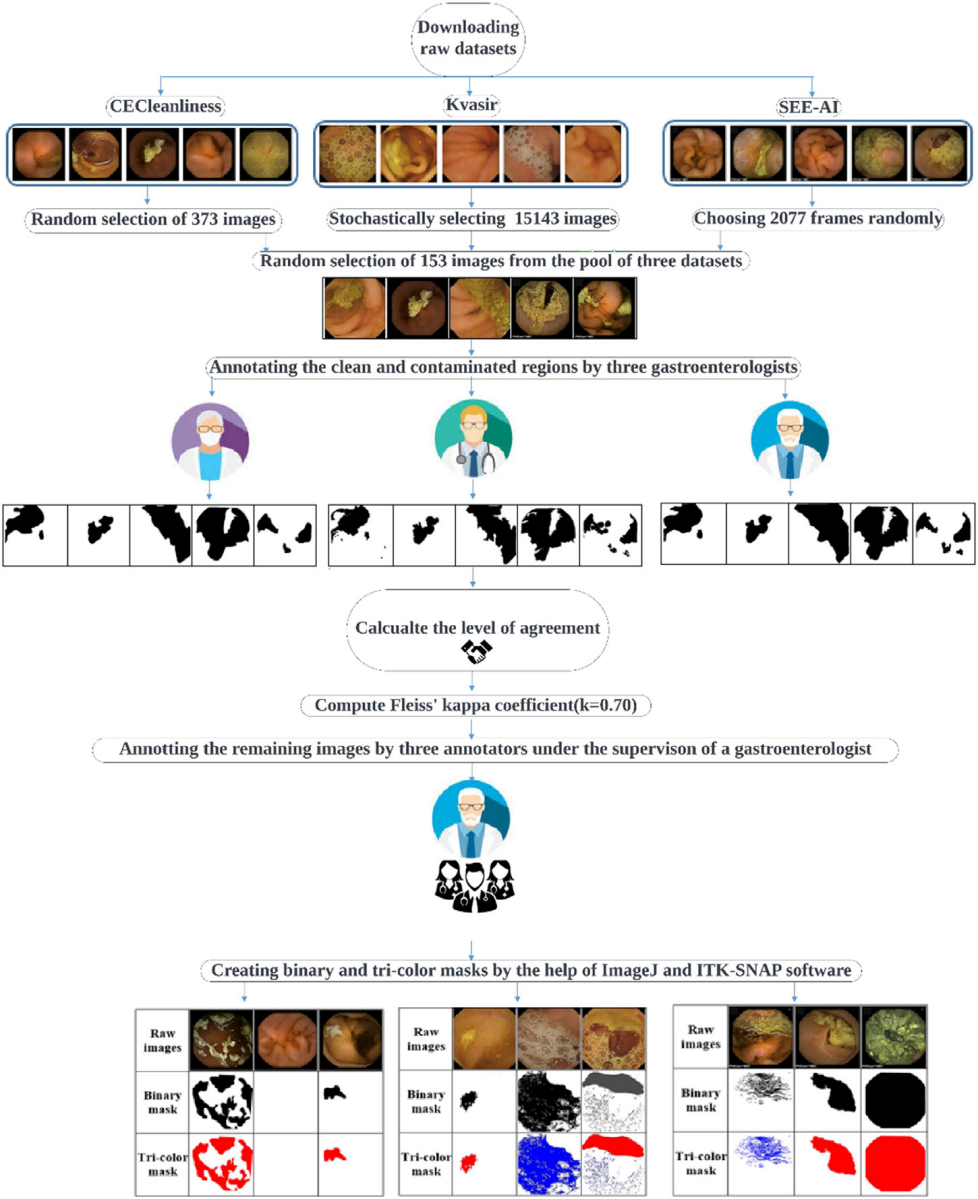
Overall flowchart of the dataset construction.

A visual workflow diagram of the dataset creation process has been depicted in Fig. 1.

Two different annotated masks have been constructed for each frame in our provided datasets. In the two-class annotation, a logical black-and-white image has been generated, in which the values of 1 (white) or 0 (black) have been assigned to the clear and contaminated pixels, respectively. In other words, white pixels in the generated binary masks represent clear areas where no contamination was present, while the black pixels has been used to index areas contaminated by bubbles or other obstructive substances. The annotation for the binary mask was performed by trained annotators under the supervision of gastroenterologist who manually identified clear and contaminated regions. The process included reviewing each frame to accurately classify the pixels. Since the physiological meaning of small bowel contents such as bubbles and turbid fluids is entirely different, in the three-class annotation, a tri-colour mask has been generated in which the blue, red, and white colours have characterized the bubbles, turbid fluids, and clear pixels. Similar to the two-class annotation, annotators manually classified each pixel in the frames. However, in this case, they identified and labeled three distinct categories.

**Fig. 2:**
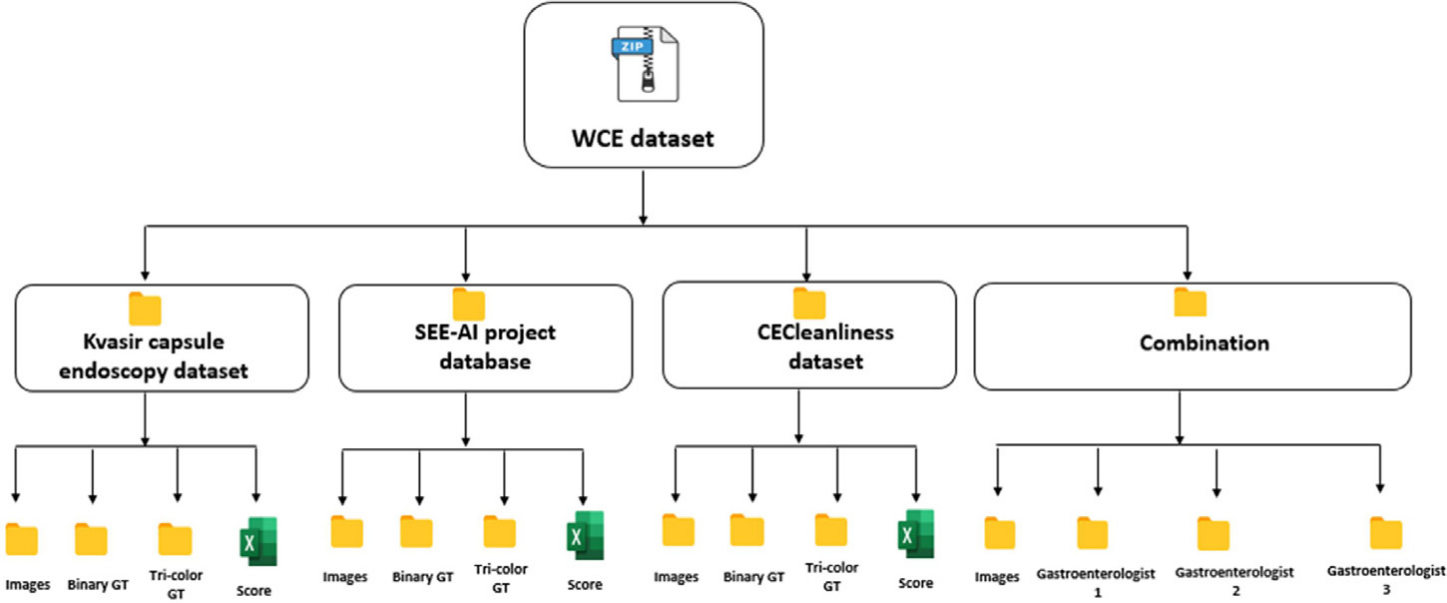
Folder structure of the current WCE dataset.

In Fig. 2 the structure of the WCE clear and contaminated region segmentation dataset is demonstrated.

The provided dataset consists of 4 main folders available in the data repository, namely “Kvasir capsule endoscopy dataset”, “SEE-AI project database”, “CECleanliness dataset”, and “Combination”.

In the “Combination” folder, there are four subfolders namely “Images”, “Gastroenterologist 1”,“Gastroenterologist 2”, and “Gastroenterologist 3”. The “Images” subfolder includes the randomly selected 153 images from the pool of raw Kvasir, SEE-AI, and CECleanliness datasets. The three remaining subfolders contain the corresponding created masks of the randomly chosen images by the three gastroenterologists.

Each one of the “Kvasir capsule endoscopy dataset”, “SEE-AI project database”, and “CECleanliness dataset” folders contain “Images”, “Binary GT”, “Tri-colour GT”, and “Score” subfolders. In each subfolder, the “Images” contains raw frames from the related capsule endoscopy dataset. The “Binary GT” and “Tri-colour GT” folders contain black-and-white and three-colour GT masks corresponding to each raw image. The “Score” subfolder in each folder includes an Excel file in which the amount of clear area in each image and its cleanliness level has been reported.

**Fig. 3:**
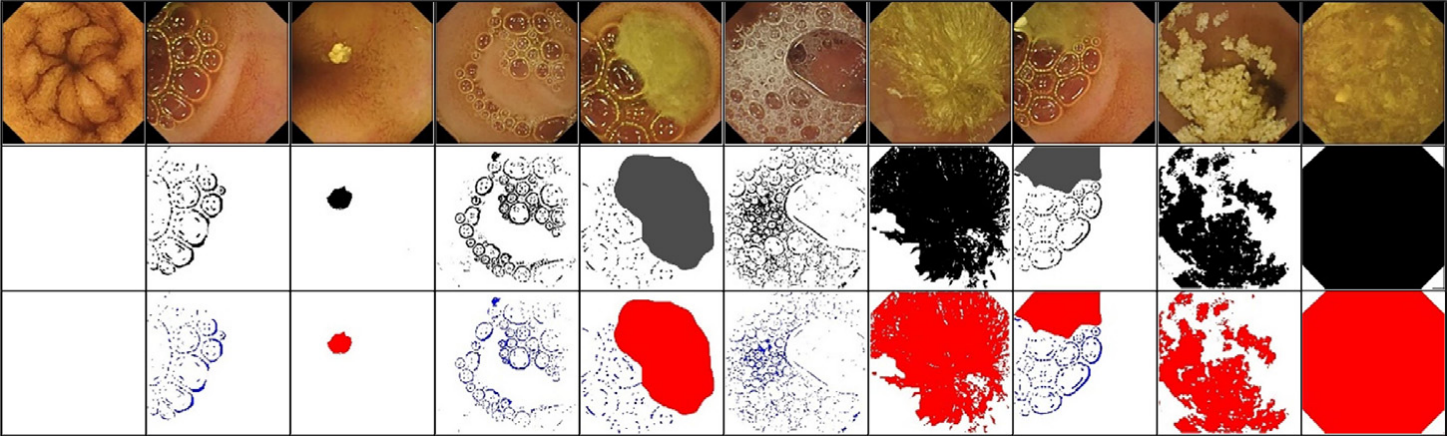
Some samples of dataset frames with their corresponding manually segmented masks. First to third rows: original images, manually annotated binary masks, and tri-variate-created GT masks.

Some sample images from the Kvasir capsule endoscopy dataset with different levels of contamination in a wide range of colours and textures with their corresponding binary and tri-colour GTs have been displayed in Fig. 3. These GT masks can be beneficial in the AI-based model training and testing. The surface of the mucosa cannot be visualized in the contaminated frames because they have been covered with different amounts of small bowel contents. As shown in Fig. 3, the clean mucosa regions behind the bubbles have been considered as clear class. Bubble-contaminated images contain condensed or scattered bubbles with various colours, sizes, and numbers. As can be inferred from Fig. 3 in the binary mask annotation (second row), the GT masks correspond to entirely clear or contaminated frames that are wholly white or black images, respectively.

In the data repository, for each one of the Kvasir capsule endoscopy dataset, SEE-AI project database, and the CECleanliness dataset, images, binary, and tri-colour masks have been converted into three distinct .npy formats using Python. These .npy format files contain the pixel matrix values of the images, black-and-white, and three-colour masks. These files can be used to carry out image processing tasks more lightly. These .npy format files can be easily opened in the Python programming language with the help of the load function from the NumPy library.

## 4 Experimental Design, Materials and Methods

In contrast to conventional endoscopies, a considerable limitation of the current WCE setup is the lack of suctioning or irrigation options because of its uncontrollable and passive movement. Due to the mentioned drawback in a normal capsule endoscopy video, with standard clinical patient preparation paradigms, from the approximately 50,000 recorded images, between 5 % to 40 % of video frames contain intestinal content [7], and 20–30 % of the complete video may be obstructed by bubbles [11]. To the best of the author's knowledge, there is no deployed clear and contaminated regions segmentation algorithm on the capsule endoscopy reading software for rejection of contaminated regions, determining the best bowel preparation regimen, and objective evaluation of SBVQ.

In order to increase the diversity of the curated dataset in the context of patient demographics and capturing conditions, enhancing the sample size, mitigating the possible bias, generalizability improvement, and heterogeneity assessment, in addition to the Kvasir capsule endoscopy dataset, some randomly chosen images from the SEE-AI project database and the CECleanliess dataset have been used for annotation. All the raw images from three already existing databases are publicly available for research purposes.

The Kvasir capsule endoscopy dataset has been collected from a series of clinical evaluations conducted at the Department of Medicine, Bærum Hospital, Vestre Viken Hospital Trust in Norway. These examinations took place over the period spanning from February 2016 to January 2018 and have been carried out utilizing the Olympus Endocapsule 10 System, which comprises the Olympus EC-S10 endocapsule and the Olympus RE-10 endocapsule recorder [3].

The Kvasir capsule endoscopy database contains 2906 and 34,833 images for reduced mucosal view and normal classes, respectively. The original images in the mentioned dataset have not been annotated at the pixel level.

The SEE-AI project dataset was curated from a cohort of 523 patients who underwent SBCE procedures utilizing the PillCam SB 3 device (Medtronic, Minneapolis, MN, USA) at Kyushu University Hospital from September 2014 to June 2021. High-resolution JPEG images, each sized 576 pixels × 576 pixels, were extracted from the recorded video footage. The dataset comprises a total of 18,481 images, with 12,320 images depicting disease lesions and 6161 images representing normal mucosal samples exhibiting diverse mucosal backgrounds [4].

The CECleanliness dataset has been collected at Hospital Universitari i Politècnic La Fe from Valencia, consisting of 854 images with a resolution of 576 × 576 pixels, and was derived from a collection of 30 videos acquired during capsule endoscopy procedures performed by the PillCam SB 3 model [5].

In contrast to the Kvasir capsule endoscopy database, where normal and reduced mucosal view image classes have been separated, the SEE-AI and CECleanliness datasets combined the clear and contaminated images.

All 2906 frames belonging to the reduced mucosal view class and the 12,237 randomly selected images from the normal category of Kvasir capsule endoscopy dataset have been used for the following pixel-wise annotation stage. Since manually annotating the clear and contaminated regions in each image is a tedious and time-consuming, the total number of 2077 and 373 images have been stochastically selected from the SEE-AI and CECleanliness for the manual pixel-level annotation.

Metrics such as inter-rater reliability (e.g., Fleiss' Kappa) were used to assess the consistency of the annotations across different annotators. This helped in validating the quality of the masks. To this end, a validation set comprising 153 randomly selected images (114, 29, and 10 images from the Kvasir, SEE-AI, and CECleanliness datasets, respectively) has been created to evaluate the level of agreement between three different gastroenterologists for the clear and contaminated region segmentation.

The WCE image annotation process begins with setting some protocols between three gastroenterologists (M.Sh., A.R., M.N.) and announcing them to the three annotators (V.S., Y.S., M.B.). For example, the black areas close to the four corners of the captured images, or the dark regions available in the WCE frames due to the absence of enough light, and the visible regions behind the large bubbles must be considered as clear class in all GT masks.

The initial annotation of the images has been meticulously carried out by three highly skilled annotators according to the established protocols. Subsequently, the annotated regions within each individual frame underwent verification, and any necessary refinement was conducted by the specialist.

**Fig. 4:**
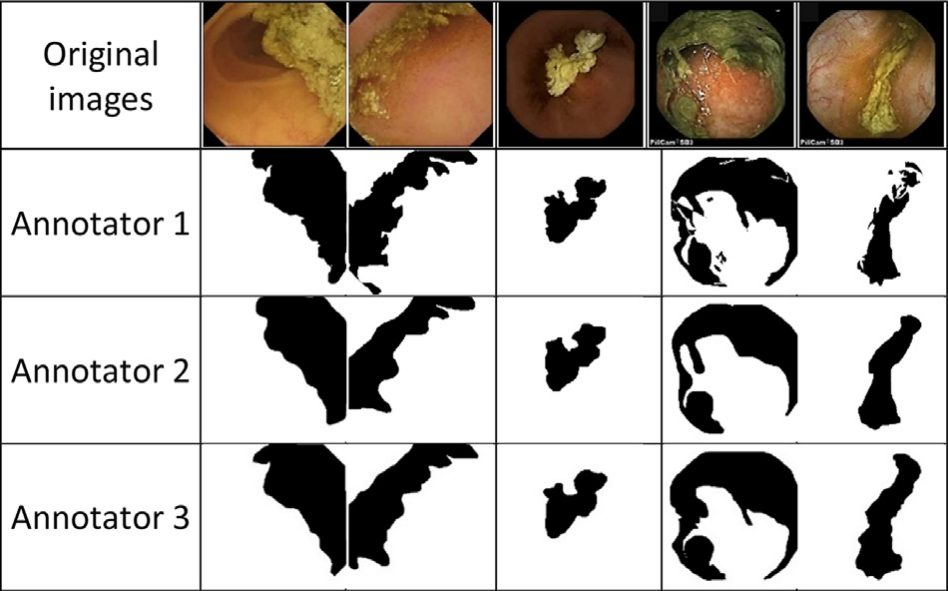
Some raw images from the three different databases along with their corresponding manually generated binary mask.

Some typical images from the pool of three raw databases, along with corresponding generated masks by three annotators under the supervision of three gastroenterologists, have been displayed in Fig. 4.

In the context of binary pixel-wise WCE image annotation by three different gastroenterologists, where each pixel has been indexed as clear or contaminated, Fleiss' Kappa as a statistical measure has been used to assess the level of agreement, evaluate the consistency and reliability of annotations [12].

The procedure of Fleiss' Kappa coefficient calculation for binary pixel-wise image annotation by three annotators has been explained in details as follows:

1. **Setup:** Consider a set of 153 randomly selected images from three different databases where each pixel needs to be annotated by three annotators, and the pixels in each image must be labelled as clear or contaminated.
2. **Construction of**N×2**matrix:** Create a matrix from all of the 153 randomly selected image where rows represent pixels and columns represent the categories (clear and contaminated). The cell values represent the frequency of each category assigned by annotators to each pixel.
3. **Calculation of observed agreement**(P)**:** Calculate the observed agreement (P) by averaging the proportion of times the annotators agree on the binary label assignment for each pixel across all images pixels. This is the proportion of agreement observed beyond chance.
4. **Calculation of expected agreement**$\;(P_{e})$**:** Estimate the expected proportion of agreement ($P_{e}$) based on the marginal proportions of each category across all pixels. This represents the agreement expected by chance.
5. Calculation of Fleiss’ Kappa (κ): Finally, Fleiss' Kappa coefficient (κ) is calculated using the formula:
  (1) $$\kappa =\frac{P-P_{e}}{1-P_{e}}$$

The resulting value of κ ranges from −1 to 1. A value of 1 indicates perfect agreement, 0 indicates agreement equivalent to chance, and negative values indicate agreement worse than chance.

To evaluate the inter-rater reliability of the annotated dataset, Fleiss' kappa coefficient has been calculated across the 153 capsule endoscopy images annotated by three independent gastroenterologists. The mean Fleiss' kappa value obtained was 0.70, with a standard deviation (std) of 0.10.

A Fleiss' kappa value of 0.70 indicates substantial agreement among the annotators, as per the commonly accepted kappa interpretation. This result falls within the range of 0.61–0.80, signifying a generally high level of consensus among the three gastroenterologists. Such a degree of agreement is particularly noteworthy given the subjective nature of medical image annotation, where variability can often arise due to individual interpretations of ambiguous regions [13].

The relatively low standard deviation of 0.1 suggests that the level of agreement was consistent across the dataset, with only minor fluctuations. While the majority of the images exhibited strong concordance in the annotations, a few images may have presented challenges due to inherent ambiguities, resulting in slightly lower agreement for those borderline cases. Nevertheless, the overall substantial agreement confirms the reliability of the dataset for segmentation tasks.

Clear and contaminated pixel-level annotation in the WCE images is a time-consuming and labour-intensive process. Annotators under the supervision of gastroenterologists need to precisely label each pixel, which requires significant manual effort and concentration. Due to our limited resources and the substantial level of agreement between three gastroenterologists (κ=0.70), one gastroenterologist, based on his accessibility, has been chosen for the following annotation process.

Achieving a Fleiss' Kappa coefficient of 0.70 suggests that the annotations made by the three gastroenterologists are reliable and consistent. Substantial agreement at this level is critical for ensuring the quality of the dataset, as it reduces the risk of error and biases. Given the labor-intensive nature of pixel-level annotation in WCE images, this high level of agreement justifies our decision to have one gastroenterologist, based on their availability, continue with the remaining annotations. This approach allows us to optimize resources while maintaining the scientific rigor and reliability of the dataset, which is essential for future model training and analysis.

The stochastically selected raw images have been randomly distributed among different annotators. To ensure the accuracy of the annotated masks, a quality control process has been carried out. Throughout the annotation process, the gastroenterologist provides supervision, oversight, and feedback to each individual annotator. The gastroenterologist reviews different subsets of annotated images periodically, offers guidance on challenging cases, and ensures that the annotations accurately represent the presence of clear and contaminated regions in the WCE images. The annotated images are reviewed iteratively to refine the annotations and address any remaining discrepancies. Any disagreements in annotations were addressed through discussions and re-evaluation. This step ensured that the final masks were consistent and reliable. In the case of challenging images the process may involve additional rounds of annotation, consensus building, and review until satisfactory agreement is reached between each different annotator and the supervisor gastroenterologist.

ImageJ and ITK-SNAP software have been used as image annotation tools in our dataset creation process since they are open source, freely available, multi-platform, and widely accepted within the scientific community.

The ImageJ software is well-suited for highlighting solid contaminated patterns by manually tracing the border of the contaminated regions. On the other hand, the ITK-SNAP as a specialized tool complements the functionality of the ImageJ software. It plays a vital role in segmenting the scattered contaminated regions such as bubbles.

The stages involved in annotating contaminated regions by using ITK-SNAP software are as follows:

1. Open the WCE image in the ITK-SNAP software using a Generic ITK Image file format, expand the view to occupy the entire window, and from the Edit menu and Slice Annotation drop-down submenu, choose the Toggle All Annotations option.
2. Highlight the contaminated regions by manually changing the image contrast from the Contrast Adjustment option in the Image contrast submenu in the Tools menu bar.
3. Remove the undesirable parts using Paintbrush Mode by setting Label Editor and Overall label opacity to [0, 0, 0] and 100, respectively. A smaller brush thickness must be used to erase delicate parts.
4. Save a screenshot of the grayscale current slice view.
5. Binarize the stored image as foreground (contaminated) and background (clear) by using the Otsu thresholding algorithm in the OpenCV Python library.

**Fig. 5:**
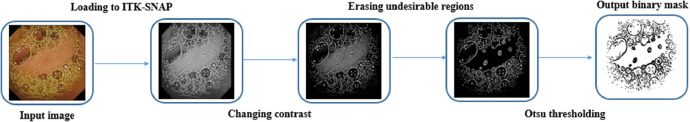
Bubble-contaminated regions annotation by utilizing the ITK-SNAP software.

The overall roadmap for annotating contaminated regions with the aid of ITK-SNAP software has been shown in Fig. 5.

To mark the contaminated regions using the ImageJ software, the image must be opened; subsequently, the contaminated regions should be highlighted by choosing the Freehand selections option in the toolbar. Finally, the binary GT mask will be constructed by right-clicking on the selected area and choosing the Create Mask option.

**Fig. 6:**
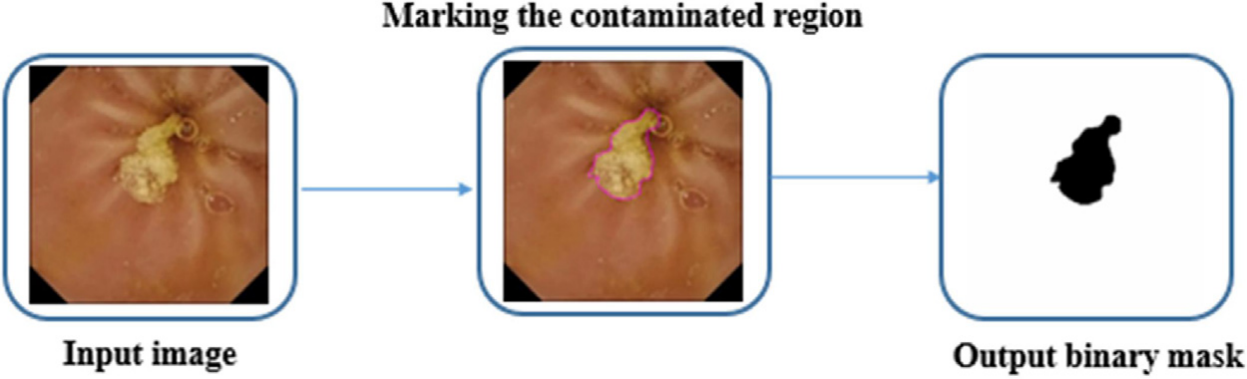
Schematic of contaminated region segmentation with the aid of ImageJ software.

The dataset generation workflow by using the ImageJ as annotation software can be observed in Fig. 6.

Each annotated image has been indexed from level 1 to 10 (from the smallest contaminated region to the largest one) from 0 to 100 % with step size equal to 10 % based on covered contaminated area in its corresponding GT mask.

The contaminated percentage on each individual image has been calculated by the following formula:

(2) $$contaminated\;area\;percentage=\frac{number\;of\;black\;pixels\;in\;the\;binary\;GT\;mask}{total\;number\;of\;pixels\;in\;the\;image}*100$$

The contaminated area percentage for each level has been computed by calculating the mean and standard deviation of clean size on the masks belonging to each level. In stage 1 a tiny portion of the image has been covered by the contaminated agents (bubbles or turbid fluids). On the other hand in stage 10 approximately entire of the image has been covered by contaminated components. In Table 1 from each visualization scale one randomly selected image along with its binary mask have been depicted. The size distribution of the contaminated mucosa area in the curated Kvasir capsule endoscopy dataset for different stages have been reported in the forth column. The last column indicates the number of images for each level in the Kvasir capsule endoscopy dataset.

**Table 1:** Some typical WCE images and their corresponding GT mask for different visualization level statistics.

Level	Example raw image	Binary GT mask	Contaminated area percentage in binary masks (mean ± std)	Contaminated area percentage in tri-color masks		Number of images
				Bubbles (mean ± std)	Turbid fluids (mean ± std)	
1			0.28 ± 1.29	2.43 ± 2.63	2.18 ± 2.84	13,023
2			14.18 ± 2.96	4.14 ± 4.96	10.62 ± 6.29	567
3			24.35 ± 2.75	5.23 ± 7.37	20.34 ± 7.73	463
4			33.65 ± 2.87	9.28 ± 13.20	25.64 ± 12.99	303
5			44.50 ± 2.97	8.58 ± 13.74	37.47 ± 14.27	223
6			54.60 ± 2.87	5.08 ± 11.80	50.74 ± 12.45	223
7			63.65 ± 2.74	7.63 ± 16.02	57.45 ± 16.34	85
8			74.39 ± 2.94	2.27 ± 1.96	73.15 ± 3.64	58
9			84 ± 2.35	1.90 ± 0.99	83.13 ± 2.73	22
10			95 ± 0.0	0.0 ± 0.0	95.0 ± 0.0	176

As can be observed in Table 1 the size of the contaminated regions increases linearly with the stage number.

**Fig. 7:**
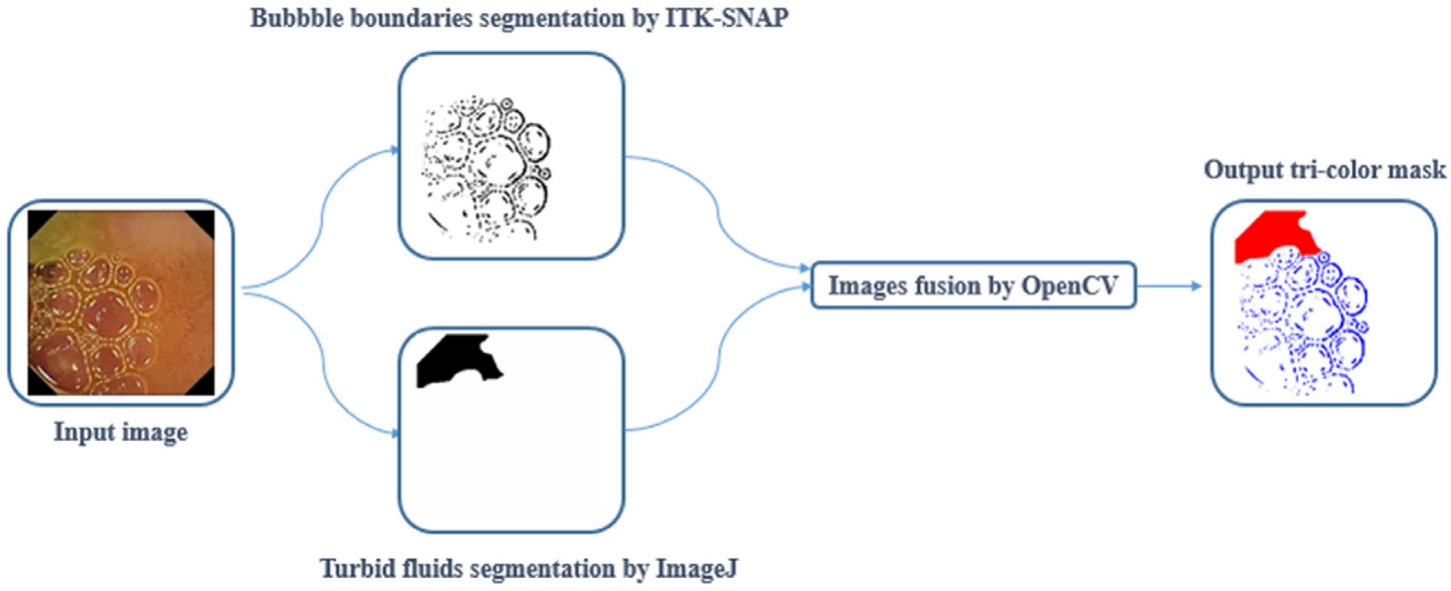
Block diagram of creation tri-colour GT mask.

To generate tri-colour masks depicting bubbles, turbid fluids, and normal tissue in images where all three components are present, we employed a combination of ITK-SNAP and ImageJ software. Initially, bubble-contaminated areas were annotated using ITK-SNAP, while ImageJ was utilized to annotate condensed turbid fluids contaminated areas. Subsequently, to create the tri-colour mask for such images, the black regions corresponding to bubble-contaminated regions and small bowel contents were modified to blue and red, respectively, utilizing the OpenCV library. The procedure of three-class segmentation mask construction can be observed in Fig. 7.

The final dataset includes a comprehensive set of images with two-class and three-class annotated masks. The dataset is structured to support various analytical and model training tasks. The annotated masks have been stored in .jpg, and .npy files and are managed through Mendeley data repository.

## Limitations

In addition to the mentioned limitations related to the WCE setup, we faced several challenges during data creation procedure and images annotation process.

Dataset was created using images from only two capsule endoscopy systems (Endo Capsule and Given Imaging). This limited scope may not fully capture the variability present in images from other brands or WCE devices, potentially affecting the generalizability of the dataset. The annotated dataset may have imbalances in the representation of different types of contamination (bubbles and turbid fluids) or clear regions.

In the data annotation procedure, ambiguous and borderline cases presented significant difficulties, as distinguishing between clear and contaminated regions was not always straightforward. These cases are characterized by images where the distinction between clear and contaminated regions is unclear due to overlapping features or subtle variations. Annotators often experience difficulties in these instances, such as inconsistencies in identifying the exact boundaries of contamination. To address these challenges, consensus meetings between each annotator and the gastroenterologist has been held to reach an agreement on challenging cases.

The presence of ambiguous annotations can affect the quality of the dataset. These ambiguities may introduce variability in the labelled data, potentially influencing the performance of computer vision-based models training on it. Models may struggle to generalize effectively or exhibit reduced performance on borderline cases.

Looking ahead, there are opportunities for future improvements in the annotation process. Enhanced annotation tools, and advanced artificial intelligence-based algorithms, could be incorporated to assist in identifying the boundary of contaminated regions in the ambiguous cases in an objective way. Additionally, the need for a broader dataset that includes images from various manufacturers is crucial for making the annotations more robust and generalizable.

## Ethics Statement

We did not need permission or ethical clearance for using the images that we used.

## CRediT Author Statement

**V.S.:** Writing – original draft, Writing – review & editing, Data curation, Software; **Y.S.:** Data curation, Software; **M.B.:** Data curation, Software; **A.V.:** Conceptualization; **M.Sh.:** Conceptualization, Data curation, Software, Supervision; **A.R.:** Conceptualization, Data curation, Software, Supervision; **M.N:** Conceptualization, Data curation, Software, Supervision; **A.M.:** Conceptualization, Project administration, Writing – original draft, Writing – review & editing.

## Data Availability

Pixel-wise AnnotatPixel-wise Annotation for Clear and Contaminated Regions Segmentation ion for Clear and Contaminated Regions Segmentation in Wireless Capsule Endoscopy Images: A Multicentre Database (Original data) (Mendeley Data).
